# Novel Neurorights: From Nonsense to Substance

**DOI:** 10.1007/s12152-022-09481-3

**Published:** 2022-02-08

**Authors:** Jan Christoph Bublitz

**Affiliations:** Faculty of Law, Universität Hamburg, Rothenbaumchaussee 33, 20148 Hamburg, Germany

**Keywords:** Neuroscience, Human rights, Neurorights, Neuroessentialism, Regulation

## Abstract

This paper analyses recent calls for so called “neurorights”, suggested novel human rights whose adoption is allegedly required because of advances in neuroscience, exemplified by a proposal of the Neurorights Initiative. Advances in neuroscience and technology are indeed impressive and pose a range of challenges for the law, and some novel applications give grounds for human rights concerns. But whether addressing these concerns requires adopting novel human rights, and whether the proposed neurorights are suitable candidates, are a different matter. This paper argues that the proposed rights, as individuals and a class, should not be adopted and lobbying on their behalf should stop. The proposal tends to promote rights inflationism, is tainted by neuroexceptionalism and neuroessentialism, and lacks grounding in relevant scholarship. None of the proposed individual rights passes quality criteria debated in the field. While understandable from a moral perspective, the proposal is fundamentally flawed from a legal perspective. Rather than conjuring up novel human rights, existing rights should be further developed in face of changing societal circumstances and technological possibilities.

The idea of neurorights, proposed novel human or fundamental rights that address matters arising from neuroscience, has recently gained attention among ethicists, politicians, and in the press. Individuals and initiatives have begun lobbying for these rights, and there are indications that these attempts are heard by domestic and international political organizations. However, a scholarly debate on neurorights, especially among human rights scholars, has yet to commence. So far, the concept of neurorights has not been explicated in scholarly work, it is rather used as a phrase, and sometimes more as a rallying cry. This paper wishes to provide critical impulses for a more thorough debate on neurorights. It is motivated by the worry that the way in which these rights are currently postulated, presented, and promoted may evoke impressions reminiscent of Bentham’s [1] notorious verdict about human rights: nonsense upon stilts.

This worry is exemplified by the proposal of the “Neurorights Initiative” (NRI, the “Initiative”), the most visible call for the adoption of neurorights (the “proposal”) that adherents actively lobby for and that receives coverage in the world press. According to its website, the NRI “works to incorporate the five NeuroRights or equivalent protections into existing human rights documents throughout the world”.^1^ A paper, loosely connected to the initiative, and a comment on the proposal were prestigiously published in the journal Nature [2, 3].^2^ The demand for neurorights is presented to international institutions such as the Inter-Parliamentary Union [4]. Significantly, advice of the initiative apparently led the legislature of Chile to amend the Chilean Constitution and to adopt a “Neurorights bill” in December 2020. The NRI lists this as an “achievement” at their website. For lack of familiarity with Chilean law, I am unable to comment on whether this amendment is, or the process that brought it about was, expedient under the domestic Chilean conditions.^3^ It should be noted, however, that amending Constitutions belongs to the most complex and time-consuming endeavors in lawmaking, usually preceded by years of public and scholarly debate. The Chilean case is thus the most impactful intervention with respect to neurorights so far.^4^

A few aspects should be mentioned at the outset. The question addressed in the following is neither whether novel technologies such as those emerging from advances in neuroscience may or should prompt changes in the law – they probably do; nor whether neurotechnologies may adversely interfere with human rights – they surely can. The questions are rather whether the challenges that the proposal outlines indeed exist in the way that it suggests, and whether the proposed neurorights, as a class as well as individuals, are reasonable or necessary answers to it. Moreover, there is no doubt that the work of the NRI is animated by a well-meaning humanistic spirit; the proposal may simply derive from interdisciplinary misunderstandings. Nonetheless, as the proposal exists and has real effects, it warrants scrutiny and discussion by the neuroethics and neurolaw communities by the standards of the field.

The following discussion touches upon several points. It addresses worries about novel human rights in general and about the proposed rights in particular; it critically examines premises and potential presuppositions of the proposal, the way the proposal engages with existing human rights law and the blending of scholarship and activism. The first section briefly introduces the proposed rights. The second section abstractly addresses a central worry that such calls typically evoke, the inflation and concomitant devaluing of human rights. The third section draws attention to the formal point that the proposal is not based in relevant scholarship although calls for changes of that magnitude should be so. The fourth section is a reminder that human rights draw limits to democratic decision making and thus require special justification. The fifth engages with one of the premises of the proposal, namely that current law is insufficient to deal with the new challenges and that novel fundamental rights are the solution. The subsequent section addresses two background considerations that put the proposal into context, neuroessentialism and neuroexceptionalism. The seventh section addresses the proposed individual rights in light of legal-technical quality criteria for novel human rights taken from the relevant literature. To anticipate: It will be argued that the proposed neurorights are poorly drafted and are no suitable candidates for adoption as novel rights. Consequently, lobbying on their behalf should cease. Nonetheless, neurotechnologies pose intriguing and substantive legal questions that should be addressed by interdisciplinary scholarship. Two more promising approaches are sketched in the final section.

## The Proposed Rights

The NRI has not published papers or elaborations on the rights it proposes, only some brief statements on their website and interviews in the press.^5^ But as the absence of substantive work does not seem to deter it from advising political actors, the few available material should be put to scrutiny and discussion. The following takes the proposal as what it proclaims to be: A proposal for the adoption of rights in the hard legal sense. Advertised and understood as such, it should be judged according to the relevant standards. To provide an impression of the proposal, here is the description of the rights from the Initiative’s website^6^:

The *right to personal identity*: “Boundaries must be developed to prohibit technology from disrupting the sense of self. When neurotechnology connects individuals with digital networks, it could blur the line between a person’s consciousness and external technological inputs”.

The *right to free-will*: “Individuals should have ultimate control over their own decision making, without unknown manipulation from external neurotechnologies”.

The *right to mental privacy*: “Any data obtained from measuring neural activity (“NeuroData”) should be kept private. Moreover, the sale, commercial transfer, and use of neural data should be strictly regulated.” The previously mentioned Nature paper considers the idea that a new regulation for neurodata “may be analogous to legislation that prohibits the sale of human organs” [3], 161). What sounds like an idea for discussion there apparently turned into a concrete policy advice by the NRI subsequently.^7^

The *right to equal access to mental augmentation*: “There should be established guidelines at both international and national levels regulating the development and applications of mental-enhancement neurotechnologies. These guidelines should be based on the principle of justice and guarantee equality of access to all citizens.”

*The right to protection from algorithmic bias*: **“**Countermeasures to combat bias should be the norm for machine learning. Algorithm design should include input from user groups to foundationally address bias.”

These rights touch on broad issues, every right might merit elaborate, monograph-length treatment on its own. Here, only a brief commentary on them in light of quality criteria for novel rights will be provided in the sixth section. Before, more abstract and formal worries about the proposal shall be addressed.

## The Worry of Rights Inflation and Devaluation

Let us begin with first impressions. To ears of human rights scholars, calling for no less than *five* novel rights to be added to the international lists might appear bold. Human rights are the most fundamental, abstract, and universal legal guarantees. Existing rights revolve around existential issues such as life and liberty, equal treatment, access to water. Although the lists of rights steadily grew over the last 70 years, they are still few in number. For good reasons. A central worry is the *inflation* of rights and their resulting *devaluation*. Human rights are powerful tools, they transform the legal landscape and potentially the lives of billions of people, they are “hard currency” in political struggles. If every important interest or legitimate concern became a matter of human rights, they may lose their distinction, significance, and effectiveness, it is widely feared (Tasioulas [5]). The following remark by James Griffin about human rights expresses the worry among human rights scholars: “There are strong inflationary pressures on the term that have brought about its debasement […] and they are still at work. The belief is widespread that human rights mark what is most important in morality; so whatever any group in society regards as most important, it will be strongly tempted to declare to be a human right” [6], 92). This worry of proliferation and debasement arose from innumerous attempts in last decades to lend grandeur to particularistic political goals by framing them as human rights concerns [7, 8]. The existing human rights have grown out of years of careful considerations (and often political struggles), and most still await full realization. By ever expanding the lists, their exceptional status, their demanding nature, and the categorical priority of their implementation are weakened and diluted; they are endangered to turn into symbols and empty rhetoric.^8^ From this perspective, calling for no less than five additional rights might portray an attitude of insensitivity to the nature and value of such rights.^9^

Furthermore, the proposal suggests that existing rights are insufficient to address the challenges of neurotechnologies. As we will see, it is anything but clear whether this claim is correct. But proclaiming the impotency of existing rights without engaging or interpreting them fails to take them seriously and adds to their diminution. Instead of disregarding existing rights and conjuring up novel rights, a parsimonious approach in loose analogy to Occam’s Razor should be adopted: human rights should not be multiplied without necessity. The burden of persuasion falls on the proponent.

## Scholarship or Activism?

Another abstract and formal, but not insignificant point concerns the role of scholarship. Advocates for legal reforms should be clear about the standpoint from which they speak. As private citizens, the horizon of rights one may call for is wide; as a scientist or researcher, it is narrower. Proposals invoking the authority and expertise of science and scholarship should satisfy several conditions: They should come from the relevant disciplines or be affirmatively viewed by them, or at least indicate whether and the extent to which they are backed by research in relevant fields; this entails noting where one’s special scholarly expertise ends. Such clarity is important not least because of public skepticism about the role of science in policy making, as witnessed in concerns of scientists tacitly overstepping boundaries of their field and becoming disguised politicians in contemporary debates about Covid-19 policies. Preserving the authority of scholarship requires transparency about the role of scholars and the state of relevant scholarship.

The proposal of the Initiative might evoke the impression as being grounded in science and scholarship. Embedded in the website of one of the world’s leading universities, it is clearly situated in the field of science. In fact, the proposal apparently stems from people working in neuroscience.^10^ Their input to policy questions is surely welcome and often necessary; without doubt, it is highly valuable. But at the same time, it should be clear that neuroscience is not among the primarily relevant disciplines for drafting and discussing human or constitutional rights, policy proposals, or other normative matters. These are primarily human rights, constitutional, and public law. Expertise in the *object* of regulation–neuroscience–is not to be confused with expertise in regulation. This requires familiarity with the law, e.g., statutes, norms, precedents, principles, and scholarly debates.

For the main relevant field, human rights law, it should be noted that the proposed rights are not widely known, let alone affirmed; it seems they have not even been put to discussion by peers. There are no publications elaborating the proposed set of rights or individual rights, with the exception of one–mental privacy. The idea has received some scholarly attention (e.g., [9–12], with mixed results regarding its recognition as a standalone right [13–15]. Apart from that, I am not aware of a single peer-reviewed publication in the field of human rights or constitutional law that provides a clear and substantive statement about meaning or scope of the other proposed rights.^11^ Of course, there are ongoing debates in law and policy making which might at some point motivate such rights, but they have not yet found a clear conceptualization. Accordingly, it has to be diagnosed that the proposed rights are not grounded in legal scholarship. But without some legal groundwork, doctrinal and conceptual work as well as publications and critical discussions, scholarship-based advocacy seems largely impossible.

One might suppose that values and policies are no objects of scholarship. But that would betray a misunderstanding. For instance, a range of technical considerations apply to norms, contracts, and statues; there is good and bad lawmaking. Quality criteria for novel human rights are debated in the field since the 1980ies (more below). In general, many reasons speak for and against any right, and good policy proposals lay them out, along with assessments of wider implications and consequences of the proposal. The Initiative does not provide such assessments. Should they exist, they ought to be provided to the public. After all, the proposal calls for no less than changes in human rights law and constitutions, the foundational documents establishing the architecture of the state and the relation between states and citizens.

## Human Rights Limit Democracy

One might also be under the impression that novel rights are inherently or always a good thing as they add something positive to the previous state of affairs. However, there is no such thing as a free right. By definition, legal rights impose duties, e.g., on the state. This is not limited to financial costs. Constitutional and human rights *restrict* the scope of democratic decisionmaking – that is their very point. Human rights cannot easily be overridden by a democratic majoritarian will, they regularly “trump”, in Ronald Dworkin’s [16] famous phrase, other high-ranking interests. Novel rights come with consequences attached. Three brief examples: The proposal calls for strict regulation of neurodata. But this may cause severe obstacles to research for many beneficial purposes. A strong protection of mental privacy may thwart legitimate public interests in law enforcement and the administration of justice (e.g., impairing the acquisition of evidence). Access to mental augmentation tools may lead to profound changes in competitive job markets and educational systems. These are delicate and complex issues. Their regulation would be strongly affected by the proposal. Surely this needs thorough reasoning. Taken seriously, rights are not only affirmative symbols, but powerful tools in societies governed by the rule of law.

Scholars promoting them bear some responsibility for shaping these tools. Although lawmakers remain responsible vis-à-vis the public, they hear advice from scholars precisely because they lack expertise about issues such as emerging technologies. A lawmaker informed by scholars from prestigious institutions about dangerous new technologies that “could challenge the very notion of what it means to be human”, with the “potential to foundationally alter society” better heeds their advice.^12^ Lawmakers act in an epistemic deficit that advisors fill with their expertise. This generates responsibilities. With respect to technologies, the Initiative endorses the idea of “responsible innovation”.^13^ This may also apply to legal innovations. And this entails, among others, transparency about the state of scholarship, comprehensive preparatory work as well as consequence-assessment of proposals. At best, in the bright light of the public. The proposal, as far as publicly available, might not live up to this standard.

## Law as it stands & Law as it should be

It is impossible to call for reforming the law without speaking about its current state; calls for reforms logically entail the claim that the status-quo is deficient. Such calls should therefore begin by clearly identifying the shortcomings of current law, e.g., gaps, objectionable consequences, impracticalities. In a second step, remedies and reforms can be discussed. As a rule, everyone calling for reforms of the law should explain how the law currently is, and where it supposedly falls short (a standard practice in serious legal discussions).

The proposal seems to skip this step by assuming that current law is inadequate. It explains: When “the Universal Declaration of Human Rights was adopted in 1948, the future challenges of Neurotechnology and Artificial Intelligence could scarcely be imagined. Consequently, there are no provisions in the human rights document to tackle new risks produced by technological innovations.”^14^ The former claim is correct, but the second, a premise of the proposal, does not follow. Law can apply to cases which lawmakers did not foresee because of the abstract and general nature of norms, especially of human rights. They are *designed* to capture a wide range of known and unknown of cases. The application of rights to unforeseen cases is precisely the reason why rights should be drafted very carefully and in anticipation of many potential consequences.

In general, the protection of human dignity, integrity, health, and related core aspects of the person are among the main concerns of the Universal Declaration of Human Rights (UDHR), irrespective of the means by which they might be threatened. Since the adoption of the UDHR, human rights law has continuously evolved through new covenants and treaties. The state of the law in 1948 to which the NRI refers is largely irrelevant today. Should the NRI harbor originalist inclinations, it should be noted that many treaties are considered “living documents” to be further developed in light of novel challenges through “evolutive” or “dynamic interpretation” [17]. An entire ecosphere of treaty bodies, Committees, Commissions, Rapporteurs, and other institutions monitors, comments, and further develops human rights law. Therefore, the premise that human rights law cannot provide protection against neurotechnologies because they were unforeseeable at the moment of adoption is false, both in abstract and with respect to specific rights (more in section seven). The existence of gaps at the level of human rights law which have not come to the attention of relevant stakeholders and which require closure by the proposed rights seems to be largely an allegation that is not backed by a systematic review of the law. The real – and interesting – question is whether and where existing human rights in their contemporary constructions fall short precisely, how they might be re-interpreted and further developed, and how remaining gaps can be addressed at the level of ordinary positive law and regulation.

The problematic lack of engagement with existing law reappears at the level of the proposed solutions. The suggested rights are not situated in existing human rights law, they remain disconnected to existing rights (more below). As a consequence, the proposal appears more like a lofty proclamation rather than a serious analysis of the law, or a substantive contribution to it. Because of this shortcoming, it may well lack impact. It is not easy to see which actual regulatory problem or legal case would be solved differently if the proposed rights were adopted –and whether this outcome would be better than the one under current law. What is needed are finer, well-considered and workable *policy and regulatory proposals* which are situated at a different level (*infra*).

## Essentialism & Exceptionalism

Furthermore, the proposal exhibits a feature familiar to everyone working at the intersections of the humanities and neuroscience – an overemphasis on the “neuro”. It is already manifested in the compound “neurorights”. The prime object of protection of the proposed rights are neither neurons, nor are the problems these rights address only caused by neurotechnologies, not even most of the time. Some of these rights – personal identity, freedom from algorithmic bias – do not seem to stand in any closer relation to neurons. The prefix “neuro” is thus a misnomer. Moreover, “neurorights” do not easily fit into to established categories of existing rights such as political, democratic or socio-economic rights. Most neurorights are variations of rights to the person.^15^ As the need for a novel class of rights has not been demonstrated, “neurorights” seems neither descriptively adequate nor systematically helpful.^16^

The focus on the “neuro” corresponds to a phenomenon which Stephen Morse [18] aptly called “brain overclaim syndrome”. In the last decade, every scientific discipline – even theology – heard great proclamations of neuroscience prompting dramatic revisions of the field. However, not many of them realized, most turned out to lack substance. A reason for this might be found in the assumption – sometimes called “neuroessentialism” [19] – that definite explanations for mental or behavioral events, even social phenomena, are ultimately found at the level of the brain. However, no good reasons support or necessitate this priority of the neurolevel, unless one is, in John Bickle’s phrase, a “ruthless reductionist” [20]. Strong forms of reductionism are a fine methodological position for neuroscience, which may and should try to explain as much as possible at the neural level. But one should not mistake a methodological assumption of a scientific field as a basis for ethics or public policy [21]. Without solution to the mind-brain problem, neuroreductionism does not possess any scientific or metaphysical priority over non-reductive explanations or more holistic images of humankind.

The law witnessed a first and ultimately futile wave of neuroscience-backed calls for reform with respect to the question of free will and criminal responsibility in the first decade of this century (e.g., the seminal paper by Greene and Cohen 64). Present calls for neurorights are about to become the second-round of claims overestimating the relevance of the field of neuroscience. Drawing on the lessons of the past, I wish to recall Morse’s [22] advice, namely the adoption of an attitude of “neuromodesty” to avoid “irrational exuberances of neuroscience on the law”.

A related concern is *neuroexceptionalism*, the idea that matters concerning the neurolevel are somehow exceptional, i.e., relevantly different to others. The problem with this idea is that it may insinuate categorical differences where there are grades and continuations. The proposed neurorights carry a flavor of neuroexceptionalism: What, for instance, is special about neurodata with respect to regulation, compared to various other forms of legally recognized sensitive data, such as logs of one’s Google searches, movement profiles stored by smartphones, banking data, health data? – Presumably, not much. Why then a novel distinct framework rather than embedding it in existing ones? Either one views neurodata as exceptional – then neuroexceptionalism looms –, or as another instance of sensitive data – then calls for a separate legal framework lose traction. The same question arises with respect to privacy: What is special about *mental* privacy in comparison to all the other privacy interest subsumed under the general right to privacy?

A third point concerns what one may call *neurosciencefictionism* or *neurohype* (e.g. [23, 24], the unclear distinction between immediate concerns that call for swift regulatory action, anticipated concerns that may or may not realize at some point in the future and often intermingle with science fiction, and thought experiments that draw on imaginations to illustrate a normative point. While legal regulation should of course be anticipatory, they should also be based on a realistic assessment of powers and potentials of technologies. The proposal conveys a sense of urgency that might create time pressures which curtail room for deliberation and debate. It pushes the narrative that innovations “could challenge the very notion of what it means to be human”.^17^ It is easy to evoke deep-running fears about novel technologies, but this might not be the best mindset for good policymaking. Rather than fueling such narratives and reinforcing such tendencies, scholarly advice should put them into context, sorting the science from the fiction, differentiating between results of research, underlying hypotheses, broader implications of the hypotheses if verified, and predictions about future trajectories. Good scholarship actively avoids overclaims; herein lies their genuine contribution to public debates and policy advice. Brain imaging, for instance, makes great progress, but how far are we from “reading thoughts” – in a profoundly worrisome way, not vague reconstructions of YouTube clips? [25]. When does the Initiative expect that a person’s consciousness is blended with digital networks? I suspect it will take another decade before these technologies are sophisticated enough to cause widespread human rights concerns. Time for thorough debate rather than hectic lawmaking.

## Brief Commentary on Specific Proposed Rights

Against this backdrop, let us turn to the specific rights proposed. For brevity’s sake, they will be considered in light of some criteria for novel human rights that emerged in human rights scholarship in a concededly coarse manner, but this should suffice to show their unsuitability. Since many years, the field has controversially discussed which interests merit access to the pantheon of human rights, given the worry about proliferation (*supra*). A widely endorsed position is that the list of human rights should be confined to the most important fundamental and universally applicable values. This led to debates about quality criteria for novel human rights. To give a taste, here are a few, put to discussion in an influential paper by Philip Alston (1984).^18^ Suitable candidates should, among others, reflect a fundamentally important value; be relevant throughout a world of diverse value systems; be consistent with, but not merely repetitive of, the existing body of human rights law; be capable of achieving a very high degree of international consensus; and be sufficiently precise as to give rise to identifiable rights and obligations. Let us see how the proposed rights fare in light of these criteria.

The *right to personal identity* is vague. What is the meaning of identity here? As bioethical debates have shown, the term is ambiguous [26]. It can refer to *diachronic identity* (conditions under which a person is the same at two different points in time), or to personality (character), to group membership as in identity politics, and more.^19^ It is a bit unclear what a right to personal identity in the first sense may amount to.^20^ A right to one’s personality in the second sense is clearer; it might protect against interferences with one’s personality such as reputation or character. This is a valuable right, and it is, by this name or another, fully or in parts, recognized in current law. Roughly speaking, many continental European legal systems tend to focus on rights to personality, whereas US law focuses on rights to privacy (starting with Warren and Brandeis 1890; [27, 28]. But from different angles, both capture a wide range of interferences with personality [29]. Furthermore, Article 1 of the Oviedo Convention states that parties shall “protect the dignity and identity of all human beings”. How does the proposed right relate to these existing protections of identity? The proposal mentions two aspects. First, technologies should not disrupt “the sense of self”. This is itself a complex term. Does it include, e.g., refugee children seeing their parents drowning, traumatizing them for life? Or is it about a feeling towards oneself, or conditions of phenomenal selfhood? And is the right to personal identity only about this “sense”, or also about other identity related issues?

Second, the Proposal mentions neurotechnologies connecting “individuals with digital networks”, blurring the “line between a person’s consciousness and external technological inputs”. With a nod to the Extended Mind debate [30], one may wonder whether this is not already happening all the time (smartphones). In any case, the substantive question not addressed is where boundaries between humans and machines should run and who may overstep them – everyone, no one, governments? Does the right *prohibit* people to blend with digital technologies – and is it then still a right, or rather a duty? These questions show that normative content of the proposed right is largely unclear; it might not refer to an operationalizable right at all, but rather gestures towards an – indeed interesting – new chapter in the history of human-technology interactions [31]. The proposal fails to give rise to identifiable rights and obligations.

The *right to free will* may be a feast for philosophers, but less for courts.^21^ In general, the concept of free will – whether or not its substrate exists – is debated since centuries. One may wonder which of the innumerous accounts should be relevant for the law; or should the law, as commentators hesitantly ask, develop “a consensual, minimal definition of free will”? [2]. I suggest it should not. Rather, the law should avoid adopting rights to eternally contested concepts. Precision is a virtue of lawmaking.

Regarding substance, it is claimed that persons should have “ultimate control over their own decision making”. This seemingly innocuous remark goes to the heart of the free will debate, in which “ultimate control” is precisely one of the contested notions [32]. The skeptic claim is that people never possess ultimate control, as every decision can either be traced back to a long chain of deterministically caused events, extending beyond the existence of the person,or equally uncontrollable indeterminacy comes in at some point. It would be unfortunate to import this dilemma into the law.

A more fitting characterization of the role of free will in the law is that the former is a presupposition of the latter, at least with respect to (criminal) responsibility. A presuppositional analysis may indeed reveal that legal systems are not fully protecting the factual conditions of responsibility. Therefore, the law may – as the proposal suggests – consider a *right against manipulation.* But note that the interest against being manipulated is recognized by a range of legal norms revolving around ideas such as undue influence. Still, legal protection against manipulation might be unsystematic and have loopholes. It may thus be worth to systematically analyze and possibly revise legal doctrines about manipulation. Input of the cognitive sciences is of invaluable help to this project, which will soon become complex, given the ubiquitous nature of influence and the difficulties in drawing meaningful distinctions between permissible and impermissible forms (see e.g. Coons and Weber [33]). Yet, developing such a right does not require another affirmation of the idea that people should not be manipulated, but rather a theory of what this means. Thus, a broad right to free will is almost inherently unclear, its adoption would be unfortunate and unnecessary.

The importance of a *right to mental privacy* is evident not only to lawyers. But it raises the question why it should be recognized as a standalone right (as the proposal is understood to suggest). Several international instruments protect a general right to privacy or private life. According to the standards of legal interpretation, this abstract right implies more context or domain-specific variations. In other words, mental privacy is implied by the more general right to privacy.^22^ And historically, the seminal paper on the right to privacy noted in 1890: “The common law secures to each individual the right of determining, ordinarily, to what extent his thoughts, sentiments, and emotions shall be communicated to others” (1890: 199). This sounds like privacy of mental states. Proponents of a novel, standalone right to mental privacy need to show why this reading is false; why privacy of the mind is, in principle, different to, say, the privacy of the bedroom. Without this, it seems they are merely different domains of application of a broader idea, the right to be let alone.

I wish to note that sometimes, breaking off a right from a parent right can be useful for doctrinal or symbolic purposes. But this requires compelling reasons. Furthermore, the intriguing questions about mental privacy do not concern its existence, but its scope, strength and limits; e.g., how it fares with respect to legitimate public interests in infringing privacy (law enforcement). Controversial debates about these issues are ongoing; and scholarly input to them, especially regarding neuroimaging methods, would be helpful needed. This might be a more promising field for interdisciplinary policy advise.

The suggested special protection of neurodata merits comment as it was raised at several occasions [34, 35]. Existing, internationally diverging data protection laws stipulate which data might be used under which conditions for which purposes. Calls for novel frameworks must show why and where existing regulations fail, or why neurodata is so special that it should not fall under them. For example, the European General Data Protection Regulation (GDPR) has a special category of sensitive data, including genetic and health data (Article 9). Much data about the brain (“neuro”) or stemming from medical examinations of it (neuroimaging) are covered by this category. As a consequence, processing of such data is prohibited, with enumerated exceptions. Insofar as some forms of neurodata are not covered but should be so, one may insert “neurodata” to Article 9, next to other types of data such as genetic data [36]. No need for further reforms. Surely, the GDPR and other regulations may have shortcomings, but they do not arise from the nature of neurodata, but rather from developments such as big data, or data-driven business models in which consumers voluntarily exchange their data for the use of services. These issues need to be addressed, but within existing frameworks.

Attention should be drawn to ongoing debates at many levels, e.g., the “recommendation on the protection and the use of health-related data” by the UN Special Rapporteur on the Right to Privacy [37] addresses issues of AI and Big Data. Developed proposals are on the table. The task of the day would be to engage with and possibly improve them, rather than calling for separate frameworks. Finally, the absurdity of the proposal to model a neurodata framework after regulations for organ donation needs to be mentioned. Different ontological natures of bodily organs and data, the multipliability of the latter but not the former, decisively speak against this analogy. The main concern about organ donation is commodification and financial pressure to sell parts of one’s body. This is categorically different to selling neurodata. In fact, a range of legitimate interests in the use of neurodata by private actors are conceivable. Why should, e.g., a company not buy motor cortex data from consumers to optimize their BCI-gaming software?

The *right to equal access to mental augmentation* is interesting. But is it about *providing access*, or about *equality* of access, or both? These options would lead to quite different claims. The explanation calls for “established guidelines at both international and national levels”. Fortunately, such regulations seem to exist. The most prevalent method of mental enhancement, pharmaceuticals, is regulated in large parts by three international treaties, observed by several international agencies, with local offices in every country.^23^ For medical and technological devices, different countries have different regulations (often as medical devices, Sienna [35]). The demand of the NRI seems fulfilled. The real question is again a different one, namely whether those regulations should be reformed (Maslen et al. [63]).

Insofar as the NRI’s call implies *easier* access to mental augmentation tools, it should be noted that the enhancement debate is far from an “international consensus” observant of different value systems. Transculturally, people may have reasonable disagreements about e.g. the significance of nature, the sanctity of the body, the importance of the self. In fact, one may wonder why there should be an international regulatory framework at all. Given the highly technical nature of such regulations and substantive differences in regulatory cultures, e.g.,with respect to liability law, common ground is unlikely to be found. The legitimate diversity of views on the matter also speaks for local experiments rather than unified international standards. The proposed right is thus not suited as an international human right.

Finally, *the right to protection from algorithmic bias*. Biases are often wrong and should be counteracted. But why a right only against algorithmic biases, what about those of ordinary human psychology? May we not want to trade the latter for algorithmic biases if those are less severe? More generally, artificial intelligence (AI) will raise a range of human rights concerns beyond biases. A right to explanation, to human oversight, against blackboxes, to workplace rights, a right to use AI for everyone, or conversely, a right to live an analogous (non-digital) life. Many topics need to be discussed. Conceivably, AI regulation requires international cooperation – and possibly, new legal instruments – demarcating the boundaries between AI and humans (Special Rapporteur [38]). But these AI-related problems should be discussed in the proper framework, not as an annex to neuro or biases. This would be an unfortunate framing. A good place might be the recent proposal by the European Commission [39] for an Artificial Intelligence Act. Rather than adopting the proposed right, lawmakers should foster comprehensive and much broader debates about problems and regulation of AI, and wait with implementation until issues and solutions become clearer.

 The proposed rights concededly deserve deeper treatment. But these brief remarks may suffice for a verdict in light of Alston’s criteria: The rights to personal identity and free will are too vague. This is not due to abstract formulations or vagueness at the penumbra, inherent to human rights, but to substantial unclarity about the core of these rights. In some understandings – right to personality, against manipulation – there is not much new under the sun. The same verdict applies to the proposed right to mental privacy: It is repetitive of existing rights and redundant as it is implied in the general right to privacy. The scope of the right to mental augmentation and its relation to the existing regulatory frameworks are equally unsettled. Better reasons speak for diversity in regulation rather than international framework and hence *against* the adoption of novel fundamental rights. The right to algorithmic biases points to an interesting problem, but much more substantive work about it in the context of AI regulation is required. Apart from mental privacy, none of the rights is sufficiently precise to give rise to identifiable claims in concrete cases. They are neither well-considered individually, nor situated within the existing human rights framework, they repeat some existing rights and contravene others. Their level is either too broad – free will –, or overly specific – algorithmic bias; they are overinclusive – mental augmentation –, or underinclusive, as they leave out a range of potential concerns and further rights (workplace, non-digital life).^24^ In sum, these rights do not convey the impression that they were drafted to be released into the existing landscape of norms, which they affect and with which they interact. The proposal neither refers to, nor engages with existing rights, and that sense it does not seem to take human rights seriously (perhaps because of the faulty premise that current law is silent about these matters).

After all: none of the proposed rights passes quality control according to the Alston criteria. They offer solutions for problems which may not exist in the alleged way; they seek recognition as human rights without recognizing human rights law; they tend to put symbolism before substance. The class of “neurorights” does not fit in with established rights, it is tainted by neuroexceptionalism and neuroessentialism. They tend to promote rights inflationism and curb the scope of democratic decisionmaking without substantive justification. Moreover, at best half-baked proposal are a disservice to the cause. Other suggestions for policies on neurotechnologies might be easily dismissed by lawmakers, institutions, or other stakeholders when they formed an unfavorable opinion about such ideas in virtue of the present proposal. Most importantly, well-meaning but imperfect advice is neither helpful for politicians engaging with, and acting on such advice, nor for the people they represent. I therefore wish to suggest stopping the lobbying for neurorights.

## Ways Forward

Instead, deep substantive scholarship on the many challenges neuroscience and other technologies raise for the law should be promoted. Current law may indeed not comprehensively address them. Interdisciplinary collaborations and the expertise of the Initiative are needed to advance debates about several topics at the crossroads of neuroscience and law. This could even lead to a novel international instrument one day. But it will not contain the proposed rights. There is no shortage of abstract principles. What is needed are more precise definitions of scopes and strengths of existing rights in different contexts as well as more fine-grained policy proposals, i.e. normative arrangements which wittily balance a wide-range of competing interests, with foresight and under conditions of uncertainty, generating fair, meaningful and operationalizable rules in a variety of domains. Developing them is a complex and painstaking task taken up by several initiatives at different levels in less shiny but more constructive work (e.g., the Sienna Project in the EU [35]. To put it differently: What is needed are arguments about substantive questions such as balancing governmental interests in law-enforcement with individual privacy, rather than the declaration of novel yet empty legal shells.

Generally speaking, there are two ways to advance the state of scholarship: bottom-up and top-down. Bottom up, one may attend to a concrete legal question and develop solutions at the level of ordinary positive law, more or less tailored to the case at hand. This may lead to amendments of current law or the adoption of new norms, e.g., as part of the regulating of specific technologies. In a second step, one may see if the problem or the solution generalizes and requires broader activity at higher levels (for bottom-up approaches in neuroethics see Racine [40].

For such endeavors, the ordinary methodology seems advisable. First, the problem and its variations need to be identified. Second, it must be assessed in light of the law as it stands. This often requires tedious legal scholarship at different levels and in different legal domains, such as public and regulatory law, tort and criminal law, and of course across different legal systems. This task easily exceeds the expertise of a single scholar. When statutes or norms do not directly speak to the question at hand, this does not mean there is a legal lacuna. Structurally similar questions have likely been answered under different headings (e.g. pharmaceutical enhancements, international drug control regimes). Whether these broader or analogous legal norms are applicable is a primarily a legal question; whether they *should* apply an ethical and political one.

Therefore, third, the current law, has to be ethically evaluated. While such evaluations often focus on traditional topics, e.g., of medical ethics, issues at hand are often broader and include effects of regulation on innovation, distributions of risks and liabilities. Getting these aspects in sight requires input from a broad range of stakeholders. Fourth, strategies for improvement may be developed, provided current law turns out insufficient. Often, this can be achieved most effectively by amending existing laws and regulations. If this is not possible or the problem concerns a set of related questions, one may develop ideas about novel frameworks and formulate a set of norms taking a wide range of relevant considerations into account.

Several issues addressed by the proposal lend themselves to such an analysis. One may draft a list of norms about who should access neurodata for which purposes, who should be allowed to infringe mental privacy to which end, who should have access to which neurotechnologies under which conditions. This soon turns into a complex task, given that manifold interests are to be accommodated, and that norms must correspond to higher laws. If ethical suggestions are on the table, one may seriously engage in lawmaking. Interdisciplinary advice is helpful at all these steps.

Alternatively, a top-down approach begins by analyzing how existing human rights apply to a topic and seeks plausible ways to advance them. This is how human rights evolve, they adapt and develop through the application of existing norms to new cases. This approach requires in-depth legal scholarship about existing rights, such as the right to privacy and security of the person, mental integrity, freedom of thought, and further rights to the person. Sketching how rights might evolve can be a valuable contribution of scholarship to the further development of the law, as such work is frequently hard to conduct for courts, even though they must have such implications in mind when adjudicating cases. Scholarly discussions of potential constructions of rights and implications can be a very helpful service. But they are much more complex and require more nuanced deliberations than conjuring up largely empty novel rights.

To conclude: One may say in hindsight that Bentham’s notorious criticism of the individual *droits de l'homme* of the French Declaration in 1789 has not aged well. These rights have become the founding stones of today’s international human rights system. The present, much less sophisticated criticism of neurorights might be misplaced as well. After all, the law is constantly evolving in response to technological and societal developments. But we should assure that these developments are shaped by substantive scholarly work rather than shiny declarations. The lobbying for the proposed neurorights should stop, while the debate about the valid worries that the Initiative laudably raises should surely continue.

## Footnotes

1 https://nri.ntc.columbia.edu/content/our-goals [Aug 11th, 2021, copy of file with author]. Note the bicapitalization, a stylistic feature familiar from brands or products but not ideas or rights.

2 I wish to emphasize that the paper is entitled “*ethical* priorities” and as such, perfectly legitimate. Nothing here is a criticism of that paper, and it is not clear that its authors support the NRI proposal.

3 For a critical view from Chile see Zuñiga-Fajuri et al. [43]. It should also be noted that Chile’s democracy appears to be in a vulnerable state as the country is experiencing a political crisis that led to a Referendum supporting the rewriting of the Constitution. This process is currently ongoing [44, 45].

4 Post scriptum, two relevant international documents were published: UNESCO’s International Bioethics Committee announced an upcoming report that advocates “the creation of a new set of human rights called ‘neurorights’ “ https://en.unesco.org/news/unescos-international-bioethics-committee-recommendations [January 11th, 2021]. A report commissioned by the Council of Europe draws heavily on the concept [46]. Both reports are not analyzed in the following. They exemplify the worry that the lobbying for neurorights affects international organizations.

5 During the writing of this paper, the website changed and now declares that the work of the NRI is “based on three seminal articles”. https://nri.ntc.columbia.edu/ [Aug 11th, 2021]. The listed articles are written by different authors, not all of them may endorse the claims of the NRI. Moreover, the articles contain diverging statements about some points of present interest. It is thus not possible to reconstruct a clear position of the NRI from them. As the papers may not necessarily express the position of the NRI, and the NRI may not endorse all claims in the papers, they will be referenced only sporadically in the following. The articles are [3, 13, 47, 48].

6 https://neurorights-initiative.site.drupaldisttest.cc.columbia.edu/sites/default/files/content/The%20Five%20Ethical%20NeuroRights%20updated%20pdf_0.pdf [accessed Aug 10th , 2021].

7 So a spokesperson for the initiative in an interview with the newspaper El Mundo about the consultations with the Chilean government, Oct. 25th, 2019, as translated on the initiative’s website: https://nri.ntc.columbia.edu/news/neurotechnology-will-allow-us-decipher-thoughts-and-alter-them-originally-spanish [Aug 11th, 2021]. Reports of the passed Chilean bills note that “neural data have the same status as organs”, https://nri.ntc.columbia.edu/news/unanimously-chilean-senate-approves-regular-neurorights [Aug 11th, 2021].

8 The loss of value not only pertains to the symbolic level, but perhaps even more, to their institutional enforcement. Either human rights are observed and enforced, including monitoring mechanisms, Rapporteurs, safeguards, courts adjudicating them – or they remain “in the books”, abstract ideals without practical effect. The latter is dangerous as it delegates rights to symbols, eroding their authority from within. The former requires considerable resources. In a world of scarcity, inflating human rights may thus negative impact the enforcement and thereby, the effects, of existing rights. For such institutional considerations see [49].

9 Two versions of the rights-inflationism concern should be noted. One emerges from unfavorable attitudes towards human rights in general and  consequently seeks to constrain their scope. This is not the view grounding this article. The other suspects the charge of inflationism to forestall more radical, utopian transformations of the human rights system (see [50]. This critique may have merit but does not apply to present worries about the proposal. It is not too utopian, but perhaps not utopian enough, it repackages and blows up traditional worries about the integrity of the person (*infra*).

10 Members of the NRI are not named at the website. Reference is made to an expert group of 25 people, but their fields of expertise and personal support for the proposal are unclear.

11 One paper should be mentioned here, supposedly the best about novel neuorights, by Ienca and Andorno [13]. The NRI’s website recently listed it as one of the grounds of its work [accessed Aug 11th, 2021]. The paper discusses four potential rights, which are more specific and better embedded in existing human rights scholarship than those of the NRI’s proposal. Also, the proposal by Ienca and Andorno might be more a reinterpretation or “reconceptualization” of existing rights rather than the creation of novel ones.

12 Quotes from the initiative’s website, https://nri.ntc.columbia.edu/ [accessed Aug 11th, 2021].

13 https://nri.ntc.columbia.edu/content/our-story-3 [accessed Aug 11th, 2021].

14 https://nri.ntc.columbia.edu/ [last accessed Aug 11th, 2021]. This point is repeated in Yuste/Genser/Herrmann [48], which asks, among others, whether “provisions envisioned possible coercion through technology” (p 160). Historically, drafters of the Universal Declaration in the immediate aftermath of WW II may not have envisioned neuroscience, but they were driven by concerns about external forces on the human person determining their being “by psychological pressure, by economic pressure, by every possible means of propaganda and social pressure” (Charles Malik, in the verbatim records of the Commission on Human Rights [51], 507). This reasoning easily encompasses technological access to the person. More importantly, however, is the fact that it does not matter whether drafters envisioned a particular form of interference, but whether it falls under the scope of the right, construed according to contemporary challenges. In support, consider the remark  in one of the standard commentaries: Art. 18 requires States to refrain from interferences “whether this be through indoctrination, ‘brainwashing’ influencing of the conscious or subconscious mind with psychoactive drugs or other means of manipulation” [52], 413).

15 One may point to international regulations about genes and genetic interventions; do they not provide a model for neurorights? Several international instruments were indeed adopted in response to threats by new technological developments, among others, the Oviedo Convention on Human Rights and Biomedicine by the Council of Europe (1997), the Universal Declaration on the Human Genome and Human Rights (UDHG) by the UNESCO [69], the International Declaration on Human Genetic Data by the UNESCO [70], and the Universal Declaration on Bioethics and Human Rights [68]. Key issues that they address were not covered by other rights or instruments; especially reproductive human cloning, inheritable germline interventions altering the genome of future generations, and genetic discrimination as well as eugenic practices [53]. These novel aspects pose novel legal challenges because germline interventions affect people not yet born, so that they cannot invoke rights against such manipulations. Accordingly, these instruments push the idea of the genome as a collective good, as the symbolic “heritage of humanity” (Art. 1 UDHG). This is an approach not found in previous instruments. Genetic discrimination puts serious pressure on the founding idea of human equality, and usually, personal characteristics are valid grounds for unequal treatment. Thus, these instruments address something new (while they also repeat existing standards e.g., with respect to consent and research). This is unlike threats by neuroscience, most of which seem to be adequately dealt with under existing headings such as bodily or mental integrity and related ones. Moreover, one may ask what to learn from the adoption of these instruments in hindsight. The debate around mitochondrial interventions and CRISPR-Cas9 shows that a strict and comprehensive ban might not be justifiable (for a more complex and critical discussion see van Beers [54]). The price, forgoing therapeutic interventions for many debilitating diseases, may appear too high. Presumably, an approach more accommodating towards novel technologies may prevail in the future. This also points to the inflexibility of such international instruments. In any case, the present criticism is not about any symbolic clarification of human rights, but against the proposed neurorights.

16 More generally, it should be noted that human rights are usually not about specific technologies. There are neither rights to cars, nor telephones or computers, to name some world-changing innovations (concededly, a right to the internet is under discussion, but as a basic necessity like water or shelter). Also, with respect to neurotechnologies used in medicine, it should be noted that the general rules in the field, e.g. consent or the *neminem laedere* principle, apply to them as well.

17 https://nri.ntc.columbia.edu [last accessed Aug11th, 2021].

18 It should be noted that Alston ultimately rejects the idea of fixed criteria for quality control. But this does not undermine the present criticism of the proposed neurorights.

19 Further international norms pertaining to aspects of personal identity concern individual genetic make-up, Art. 3 – Person’s Identity – of the Declaration on Human Genetic Data; the right to a legal identity or personality, the right of children to preserve their identity in Art. 8 of the Convention on the Rights of the Child.

20 The law, by the way, does so. Legal systems have no problem identifying a ninety year old demented lady as the same person who attended elementary school. Non-identity is simply not a legal issue until technologies such as mind-uploading realize.

21 The irony should not go unnoticed that neuroreductive views usually motivate calls for overcoming supposedly antiscientific beliefs in free will. This would imply that the object of the proposed right is non-existent.

22 Yuste/Genser/Herrmann [48] write that the concept of “mental privacy is not contemplated within Article 17 of the ICCPR” and that the General Comment “not only fails to mention technology, but also fails to discuss the privacy of a person’s thought” (p 160). This seems to be a misunderstanding. The relevant General Comment is No. 16, adopted by the UN Human Rights Committee [55]. These Comments are brief, general and abstract; this Comment comprises eleven paragraphs to cover a vast topic. It cannot list, nor strives to list, every potential interference, but sketches broad lines. In fact, the Comment notes that privacy is “to be guaranteed against all such interferences and attacks”, indicating a broad and inclusive scope. The Comment explicitly mentions privacy threatening technologies of that time, e.g., interception of telephonic communication, wiretapping, searches of the body, data on personal computers. Moreover, the work of the UN Special Rapporteur on the Right to Privacy largely evolves around questions posed by current technologies (see the Annual Thematic Reports, [42]). Thus, there are no indications that mental privacy is not part of the right to privacy – on the contrary, it seems an evident application.

23 UN Single Convention on Narcotic Drugs [65], Convention on Psychotropic Substances [66], Convention against Illicit Traffic in Narcotic Drugs and Psychotropic Substances [67].

24 Surprisingly, the set of neurorights does not include the rights that seem most promising and perhaps most urgently needed, i.e., a right to mental integrity complementing the legal protection of the person (e.g., [21, 41, 56]); or a right to freedom of thought or Cognitive Liberty as discussed by legal scholars for some time (e.g., [57–62]).
